# Trends in the disease burden of anxiety disorders in middle-aged and older adults in China

**DOI:** 10.1186/s40359-024-01575-2

**Published:** 2024-02-19

**Authors:** Zeng Zhi, Shi Yan, He Yijuan, Zheng Jiahuan, Jiang Xiaohan, Chen Dandan

**Affiliations:** 1 School of Health and Economics Management, Nanjing University of Chinese Medicine, Nanjing, 210023 China; 2 School of Nursing, Nanjing University of Chinese Medicine, Nanjing, 210023 China; 3 Pukou Hospital of Traditional Chinese Medicine in Nanjing, Nanjing, 211899 China; 4 Science and Education Department, Taicang TCM Hospital Affiliated to Nanjing University of Chinese Medicine, Suzhou, 215400 Jiangsu Province China

**Keywords:** Anxiety disorders, Middle-aged and older adults, Age-period-cohort analysis, Disease burden, Incidence, Disability-adjusted life years

## Abstract

**Background:**

Anxiety disorders in middle-aged and older adults are an important public health concern in China. Based on the data in the global disease burden (GDB) research database, this study evaluated and analyzed the trend of the disease burden of middle-aged and older patients living with anxiety in China in the past 30 years.

**Methods:**

The incidence and disability-adjusted life years (DALYs) data of anxiety disorders in China for individuals aged 45–89 years were collected from the Global Burden of Disease Study 2019, and the effects of age, period, and cohort on the incidence of and DALY rate for anxiety disorders were analysed using an age-period-cohort model. Because of the COVID-19 pandemic, the global disease burden research database has not been updated since 2019. However, this did not affect the analysis of future trends in this study, which combined data in the past three decades from 1990 to 2019.

**Results:**

(1) The overall age-standardised incidence rate (ASIR) and age-standardised DALY rate (ASDR) for anxiety disorders in middle-aged and older adults in China decreased by 4.0 and 7.7% from 1990 to 2019, respectively, and the ASIR and ASDR were always higher in women than in men. (2)Age-period-cohort analysis showed that the net drifts for incidence and DALY rate were − 0.27% and − 0.55% per year, respectively. For both genders, the local drifts for incidence were lower than zero in those aged 45–79 years and higher than zero in those aged 80–89 years; the local drifts for the DALY rate were lower than zero in all groups. (3) From the 1990–1994 to 2015–2019, the relative risks of anxiety disorder incidence and DALY decreased by 5.6 and 7.3% in men and 4.3 and 11.7% in women, respectively.

**Conclusion:**

The disease burden of anxiety disorders in middle-aged and older adults in China has been relieved over the past 30 years; however, recent ASDR, ASDR, period, and cohort effects have shown adverse trends. The incidence and DALY rate decreased with age in women, while men showed a trend of increasing first and decreasing afterwards.

## Introduction

Anxiety disorders are a group of relatively common mental illness that include various clinical manifestations such as panic disorder, generalised anxiety disorder, and specific phobias [1]. In comparison to the general population, patients with anxiety disorders are at a higher risk for other diseases, such as sleep disorders [2], cardiovascular diseases [3], and gastrointestinal diseases [4]. Anxiety also affects many aspects such as attentional capacity and learning, as well as stress responses in terms of cortisol levels [5], and as a result, these patients have a relatively lower quality of life, with more serious anxiety symptoms associated with poorer quality of life [6]. Due to these issues, individuals with anxiety disorders require significant medical resources from society, resulting in a heavy burden of disease [7].

The prevalence of anxiety disorders has increased in recent years. In 2019, approximately 46 million new cases of anxiety disorders were reported worldwide, an increase of 47.21% since 1990 [8]. In light of its high prevalence, comorbidity, and chronicity, the World Health Organization has ranked anxiety disorders as the ninth leading cause of health-related disability globally [9]. The current situation in China is equally worrying. Over the past three decades, rapid social development has led to a general increase in psychological stress, and anxiety disorders have become the most prevalent type of mental disorder in China [10]. Previous studies have shown that biological processes of aging may contribute to poor mental health and is a potential risk factor for anxiety in middle-aged and older adults [11]. Moreover, serious public health problems such as social isolation among older people also increase their risk of mental health problems [12]. With 28% of the Chinese population expected to be over 60 years old by 2040 [13], geriatric anxiety disorders are becoming increasingly serious. Early prevention of anxiety disorders can reduce the burden on older adults and society [14], therefore, anxiety disorders in middle-aged adults should be addressed to prevent anxiety disorders in the older adult population.

Anxiety disorders in middle-aged and older adults are primarily characterised by health anxiety [15], and research on these trends can contribute to the prevention and treatment of anxiety disorders in this population, thus promoting healthy aging. However, current research on anxiety disorders mainly focuses on teenagers, with few investigations on middle-aged and older adults. Although studies have shown a trend change in anxiety disorders over time [16], the trend in the disease burden of anxiety disorders in middle-aged and older adults in China remains unclear. Therefore, this study used data from the Global Burden of Disease Study 2019 (GBD 2019) and the Age-Period-Cohort (APC) model to explore the long-term trends in the incidence and Disability-Adjusted Life Years (DALY) rate of anxiety disorders, as well as the effects of age, period, and cohort by sex in middle-aged and older adults in China. The results are a necessary supplement to the research on the disease burden of anxiety disorders. They provide a basis for the formulation and implementation of policies to prevent and treat anxiety disorders in middle-aged and older adults in order to alleviate the burden of disease and achieve the optimal allocation of health resources.

## Materials and methods

### Data sources

Data were obtained from the GBD 2019. The GBD 2019 estimated the disease burden data in 204 countries and territories for 369 diseases and injuries and 87 risk factors from 1990 to 2019 using DisMod-MR 2.1, a Bayesian meta-regression tool to ensure the comparability of the data [17]. In the GBD 2019, Chinese data on anxiety disorders were mainly obtained from the national census, disease surveillance point systems, death cause registration reporting systems, and community representative epidemiological studies [18, 19]. In this study, data on the incidence of and DALY rates for anxiety disorders in a Chinese population aged 45 to 89 years from 1990 to 2019 were selected for analysis.

### Research methods

#### Statistical indicators

Trends, such as disease incidence and DALY rate, are relatively intuitive indicators for measuring a country’s disease burden [20]. Therefore, the relevant indicators used in this study included total Chinese middle-aged and older adult populations, crude incidence, crude DALY rate, age-standardised incidence rate (ASIR), and age-standardised DALY rate (ASDR). Standardised populations were referenced to the global population of the GBD 2019 data. The specific calculation formula for the statistical indicators is as follows [21]:

$$\textrm{ASIR}/\textrm{ASDR}\ \textrm{per}\ \textrm{100,000}=\frac{\sum {\textrm{a}}_{\textrm{i}}{\textrm{b}}_{\textrm{i}}}{\sum {\textrm{b}}_{\textrm{i}}}\times 100000$$

where *a* is the age-specific incidence/DALY rate, *b* is the age-specific population number, and *i* is age class.

#### Data analysis

This study used an the Age-Period-Cohort (APC) model to assess trends in the incidence and DALY rates of anxiety disorders in middle-aged and older adults in China, and to evaluate the impact of age, period, and cohort effects on these trends. The APC model is a common statistical method used in demography and epidemiology and can be used to analyse long-term disease trends over time [22]. The high collinearity among age, period, and cohort in the APC model can lead to unidentifiable parameters [23]. Therefore, we used a web tool (http://analysistools.nci.nih.gov/apc/) based on the R code developed by the National Cancer Institute for APC analysis to solve the problem of model identification parameters [24].

The following parameters were evaluated using the APC model: net drift, local drift, the longitudinal age curve, the period Rate Ratio (RR), and the cohort RR. Net drift is the annual percentage change in log disease rates adjusted for the nonlinear period and cohort effect. Local drift is the annual percentage change in log disease rates for different age groups. The longitudinal age curve represents the age effect and indicates the longitudinal age-specific rates in the reference cohort adjusted for period deviations and can be used to infer the impact of age on trends in anxiety disorder. The period RR represents period effects and indicates the ratio of age-specific rates in the period relative to the reference period, which can be used to infer the impact of the period effect on trends in anxiety disorders. The cohort RR represents period effects, indicating the risk of incidence/DALYs of anxiety disorders in different birth cohorts [25].

To enable the APC analysis, we divided the incidence, DALY rate, and population data into six periods at 5-year intervals, that is, from 1990 to 1994 (Median 1992) to 2015–2019 (Median 2017). The age data were also divided into nine consecutive age groups at 5-year intervals, that is, from to 45–49 years old to 85–89 years old. We divided the 14 birth cohorts into 1903–1907–1968–1972. The central age, period, and cohort groups within the respective intervals were defined as references for the APC analysis. The 65–69 years age group, 2000–2004 and 1933–1937 birth cohort were set as the reference groups in this study. Estimable functions were performed using the Wald Chi-Square test (two-sided), and *P*-values less than 0.05 were considered statistically significant. Written informed consent was obtained from the participants before the experiments. The study was approved by the committee of the ethic board of Nanjing University of Chinese Medicine and the latest revision of the Declaration of Helsinki.

## Results

### Trends in the incidence of and DALY rates for anxiety disorders in middle-aged and older adults in China from 1990 to 2019

**Fig. 1:**
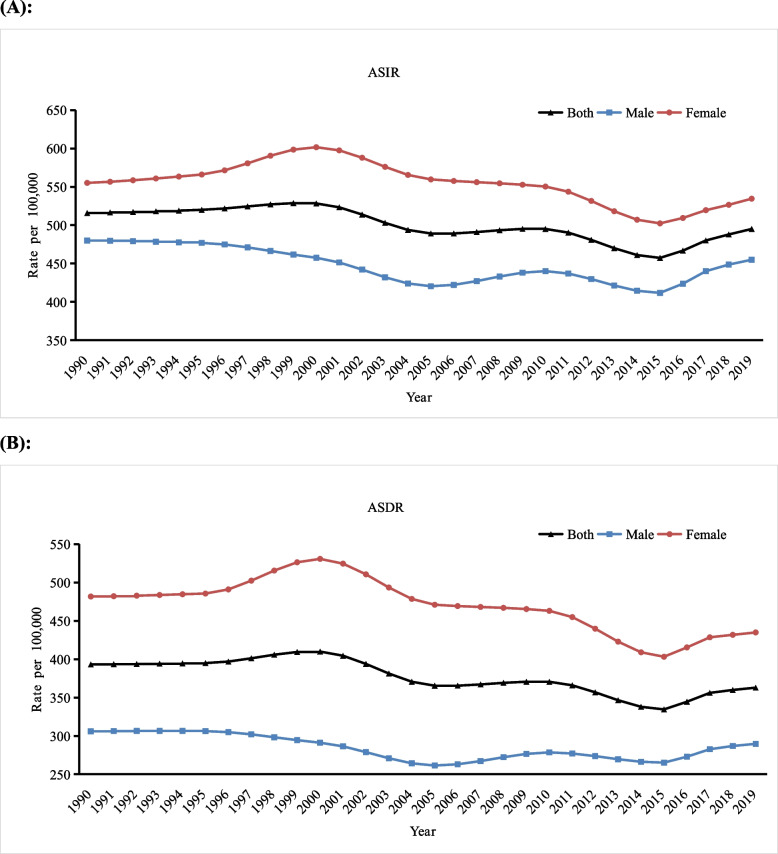
Trends in age-standardized incidence rate (ASIR) and disability-adjusted life year (DALY) rate (ASDR) of anxiety disorders in middle-aged and older adults in China, from 1990 to 2019. **a** ASIR for both genders, men, and women, (**b**) ASDR for both genders, men, and women

Figure 1 shows the trends in the ASIR and ASDR of anxiety disorders in middle-aged and older adults in China from 1990 to 2019. In the past 30 years, the ASIR and ASDR of anxiety disorders in middle-aged and older adults in China have fluctuated but are generally relatively stable. Both the ASIR and ASDR were higher in women than in men.

The overall ASIR in middle-aged and older adults in China was 495.08/100,000 in 2019, a decrease of 4.0% from 1990 (515.85/100,000). For men, the ASIR was 454.92/100,000 in 2019, decreasing by 5.2% from 1990 (479.94/100,000). In 2005, 2010, and 2015, there were three inflection points. From 1990 to 2005 and from 2010 to 2015, the ASIR in men showed a decreasing trend and reached a minimum value of 411.73/100,000 in 2015, while it showed an increasing trend from 2005 to 2010 and from 2015 to 2019. For women, the ASIR increased from 555.13/100,000 in 1990 to 601.66/100,000 in 2000 and then decreased significantly, reaching its lowest point of 502.33/100,000 in 2015. Finally, it gradually increased to 534.54/100,000 from 2015 to 2019, decreasing by a total of 3.7% over 30 years (Fig. 1a).

The overall ASDR was 362.96/100,000 in 2019, a decrease of 7.7% from 1990 (393.26/100,000). For men, the ASDR decreased by 5.4% from 1990 (306.10/100,000) to 2019 (289.69/100,000), The trend during this period was the same as the ASIR and the lowest point was 261.38/100,000 in 2015. For women, the ASDR decreased by 9.7% over 30 years. Specifically, the ASDR increased from 481.79/100,000 in 1990 to 530.89/100,000 in 2000, then decreased significantly, reaching its lowest point of 403.28/100,000 in 2015. Finally, it gradually increased to 434.95/100000 from 2015 to 2019 (Fig. 1b).

### APC model analysis of anxiety disorders in middle-aged and older adults in China from 1990 to 2019

#### Fitting of the APC model for the incidence and DALY rate in middle-aged and older adults in China

Based on the Wald Chi-Square test (Table 1), the net drifts, local drifts, age deviations, period deviations, cohort deviations, period RRs, and cohort RRs were statistically significant for the incidence and DALY rate in middle-aged and older adults in China from 1990 to 2019 (*P* < 0.05).

**Table 1:** Wald Chi-Square tests for estimable functions in the age period cohort (APC) model

Null hypothesis	Both		Male		Female	
	Chi-square	*P*-Value	Chi-square	*P*-Value	Chi-square	*P*-Value
Incidence						
Net Drift = 0	63.9	< 0.001	36.39	< 0.001	77.94	< 0.001
All Age Deviations = 0	2180.04	< 0.001	1572.67	< 0.001	2031.79	< 0.001
All Period Deviations = 0	67.72	< 0.001	124.93	< 0.001	334.39	< 0.001
All Cohort Deviations = 0	81.85	< 0.001	108.85	< 0.001	46.38	< 0.001
All Period RR = 1	126.21	< 0.001	165.84	< 0.001	394.84	< 0.001
All Cohort RR = 1	617.23	< 0.001	503.87	< 0.001	593.31	< 0.001
All Local Drifts = Net Drift	76.09	< 0.001	100.81	< 0.001	43.34	< 0.001
DALY rate						
Net Drift = 0	463.59	< 0.001	84.2	< 0.001	1041.94	< 0.001
All Age Deviations = 0	44.65	< 0.001	49.81	< 0.001	109.15	< 0.001
All Period Deviations = 0	156.58	< 0.001	152.61	< 0.001	865.27	< 0.001
All Cohort Deviations = 0	116.52	< 0.001	73.44	< 0.001	139.57	< 0.001
All Period RR = 1	607.43	< 0.001	240	< 0.001	1878.93	< 0.001
All Cohort RR = 1	950.93	< 0.001	325.72	< 0.001	1935.04	< 0.001
All Local Drifts = Net Drift	105.53	< 0.001	65.9	< 0.001	130.35	< 0.001

*DALY disability-adjusted life year: *RR* rate ratio

**Fig. 2:**
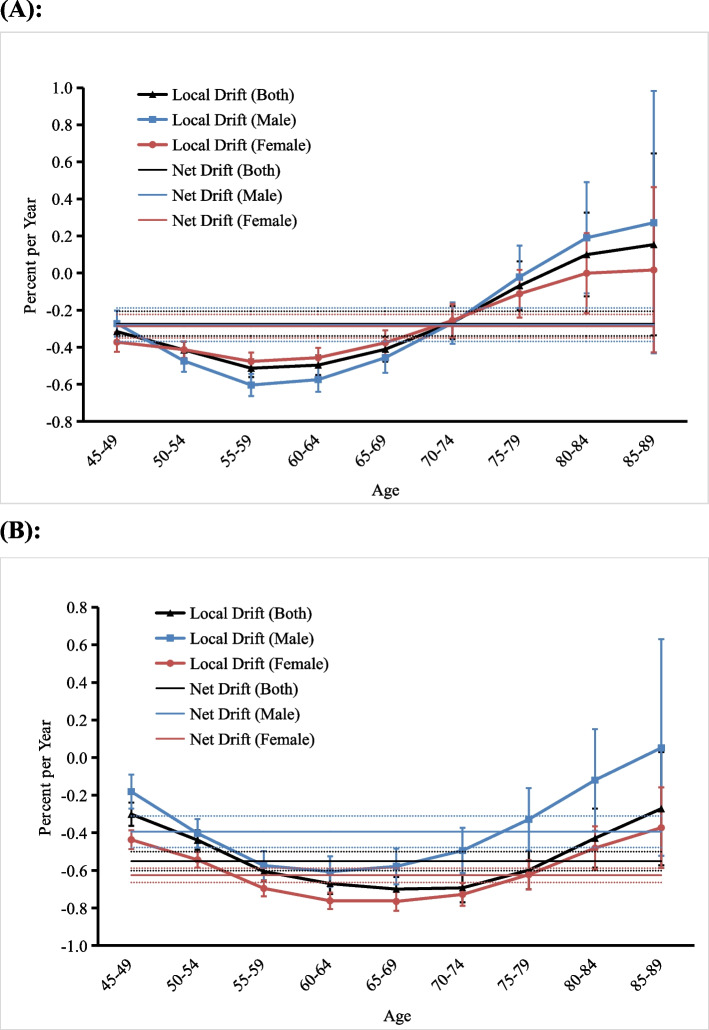
Net drifts and local drifts for the incidence of and disability-adjusted life year (DALY) rate for anxiety disorders in middle-aged and older adults in China. **a** Incidence, (**b**) DALY rate. The annual percentage change of the expected age-adjusted rates (net drift) and age-specific rates (local drift) in the incidence of and DALY rate for anxiety disorders and the corresponding 95% confidence intervals (*note:* some of them were too narrow to be shown in the figure)

Figure 2 shows the annual percentage change in the expected age-adjusted and age-specific rates of anxiety disorders over time in middle-aged and older adults in China. As shown in Fig. 2a, the net drift of incidence was − 0.27% (95% CI[− 0.34, − 0.21%]) (< 0) for both genders, with − 0.28% (95% CI[− 0.37, − 0.19%]) (< 0) for men and − 0.29% (95% CI[− 0.35, − 0.22%]) (< 0) for women. The local drifts of the incidences for both genders, men and women, showed a decreasing trend in the 45–59 years age group, reached a minimum at the 55–59 years age group, and then showed an increasing trend in the 60–89 years age group. However, the magnitude of the change was greater in men than in women. For both genders and men, the local drifts for the incidence were below 0 in the 45–79 years age group and above 0 in the 80–89 years age group. For women, the local drifts were below 0 in the 45–84 years age group and above 0 only in the 85–89 years age group.

As shown in Fig. 2b, the net drifts of the DALY rates were − 0.55% (95% CI[− 0.60, − 0.50%]) (< 0) for both genders, with − 0.39% (95% CI[− 0.48, − 0.31%]) (< 0) for men and − 0.63% (95% CI[− 0.66, − 0.59%]) (< 0) for women. The local drifts of the DALY rates were higher in men than in women in all age groups. For both genders and women, local drifts were below 0 in all age groups. They tended to decrease in the age group from 45 to 69 years, reached a minimum in the 65–69 years age group, and then tended to increase in the 70–89 years age group. For men, the local drifts showed a decreasing trend in the 45–64 years age group, reached a minimum in the 60–64 years age group, and then increased in the 65–89 years age group. The local drifts of the DALY rates in men were below 0 in the 45–84 years age group and above 0 only in the 85–89 years age group.

#### Age effects

**Fig. 3:**
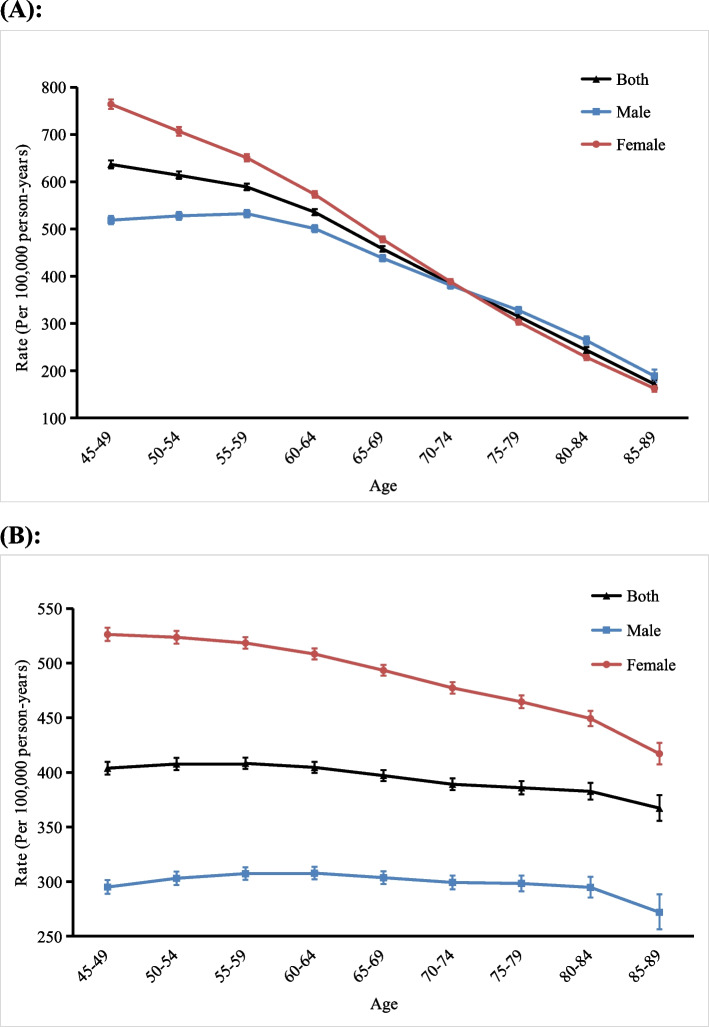
Longitudinal age curves of the incidence of and disability-adjusted life year (DALY) rate for anxiety disorders in middle-aged and older adults in China. **a** incidence, (**b**) DALY rate. Fitted longitudinal age-specific rates of the incidence/DALY rate of anxiety disorders (per 100,000 person-years) and the corresponding 95% confidence intervals (*note:* some of them were too narrow to be shown in the figure)

Figure 3 shows the longitudinal age curves for the incidence and DALY rates of anxiety disorders in middle-aged and older adults in China by sex. As shown in Fig. 3a, after correcting for period and cohort effects, the longitudinal age curve of the incidence of anxiety disorders in both genders showed a decreasing trend with age. It decreased from 636.59/100,000 in the 45–49 years age group to 171.45/100,000 in the 85–89 years age group, and the decrease significantly accelerated after the age of 60 years. The incidence of anxiety disorders in men tended to increase first and then decrease with age, reaching a maximum in the 55–59 years age group (532.30/100,000) and a minimum in the 85–89 years age group (188.62/100,000). The incidence of anxiety disorders in women showed a decreasing trend in all age groups, from 763.98/100,000 in the 45–49 years age group to 162.39/100,000 in the 85–89 years age group, and the magnitude of the decrease was relatively stable. By comparison, it was found that the incidence was higher in women than in men in the 45–74 years age group, while the incidence was lower in women than in men in the 75–89 years age group.

The longitudinal age curves of the DALY rates of anxiety disorders were all relatively smooth, and the DALY rate was higher in women than in men in all age groups (Fig. 3b). For both genders, the DALY rate showed an increasing trend in the 45–59 years age group and a decreasing trend in the 60–89 years age group, with a small magnitude of change. The age group with the highest DALY rate was the 55–59 years age group (408.24/100,000). For men, the DALY rate tended to increase in the 45–64 years age group and decrease in the 65–89 years age group, with the highest DALY rate in the 60–64 years age group (307.76/100,000). For women, the DALY rate decreased gradually with age, from 526.30/100,000 in the 45–49 years age group to 417.09/100,000 in the 85–89 years age group.

#### Period effects

**Fig. 4:**
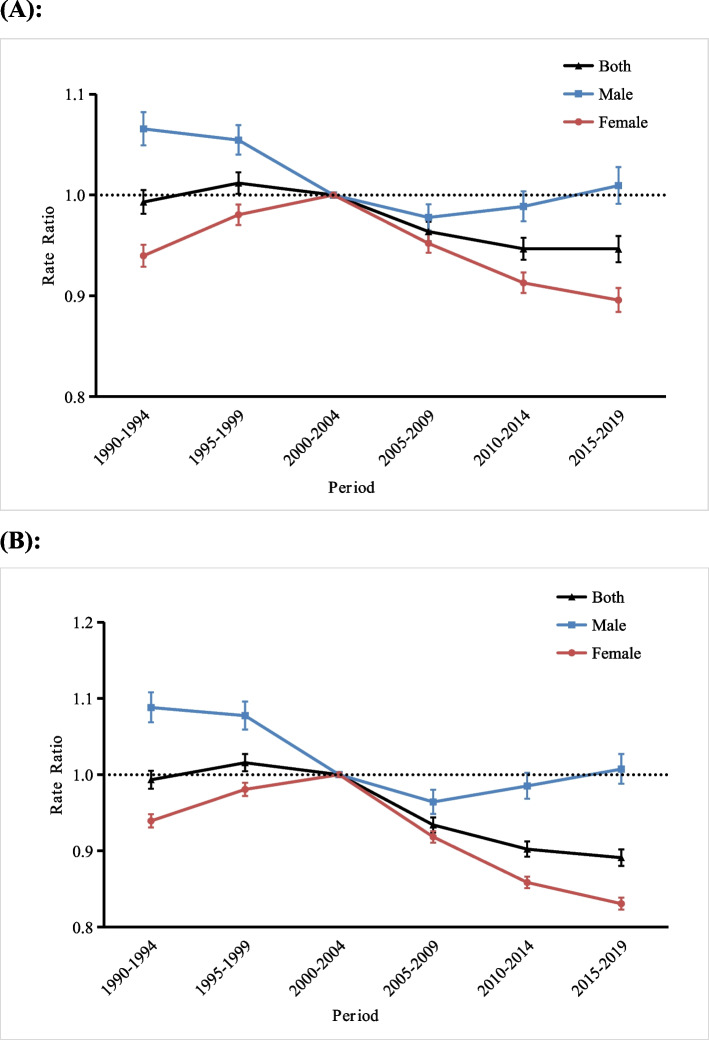
Period rate ratios (RR) of the incidence of and disability-adjusted life years (DALY) rate for anxiety disorders in middle-aged and older adults in China. **a** incidence, (**b**) DALY rate. The ratio of age-specific rates in each period relative to reference period (2000–2004) and the corresponding 95% confidence intervals. The black dash lines indicate an RR = 1

Figure 4 shows the RRs of the incidence of and DALY rates for anxiety disorders in middle-aged and older adults in China by sex throughout the observation period. The trends in the effects of period on the incidence and DALY rates were similar. The period effects for the incidence and DALY rate showed an increasing trend from the 1990–1994 period to the 1995–1999 period for both genders, with the period RR increasing from 0.99 [95% CI (0.98,1.00)] and 0.99 [95% CI (0.98,1.01)] to 1.01 [95% CI (1.00,1.02)] and 1.02 [95% CI (1.00,1.03)], and then gradually decreasing to 0.95 [95% CI (0.93,0.96)] and 0.89 [95% CI (0.88,0.90)] from the 1995–1999 period to the 2015–2019 period.

For middle-aged and older adults of different genders, the risk of incidence of and DALY rate for anxiety disorders showed different trends over the study period. For men, the risks of incidence and DALY rates tended to decrease first and then increase over the study period. The period RRs of incidence and DALY rate decreased from 1.07 [95% CI (1.05,1.08)] and 1.09 [95% CI (1.07,1.11)] in the 1990–1994 period to 0.98 [95% CI (0.96,0.99)] and 0.96 [95% CI(0.95,0.98)] in the 2005–2009 period, respectively, and then the period RRs of incidence and DALY rate both increased to 1.01 [95% CI (0.99,1.03)] from to the 2005–2009 period to the 2015–2019 period. However, for women, the risks of incidence and DALY rate tended to increase first and then decrease over the period, with the period RRs of incidence and DALY rate both increasing from 0.94 [95% CI (0.93,0.95)] in the 1990–1994 period to 1 in the 2000–2004 period, then decreasing to 0.90 [95% CI (0.88,0.91)] and 0.83 [95% CI(0.82,0.84)] in the 2015–2019 period, respectively. From the 1990–1994 period to the 2015–2019 period, the risk of anxiety disorder incidence decreased by 4.0, 5.6, and 4.3% in both genders, men, and women, and the DALY rate decreased by 10.1, 7.3, and 11.7%, respectively.

#### Cohort effects

**Fig. 5:**
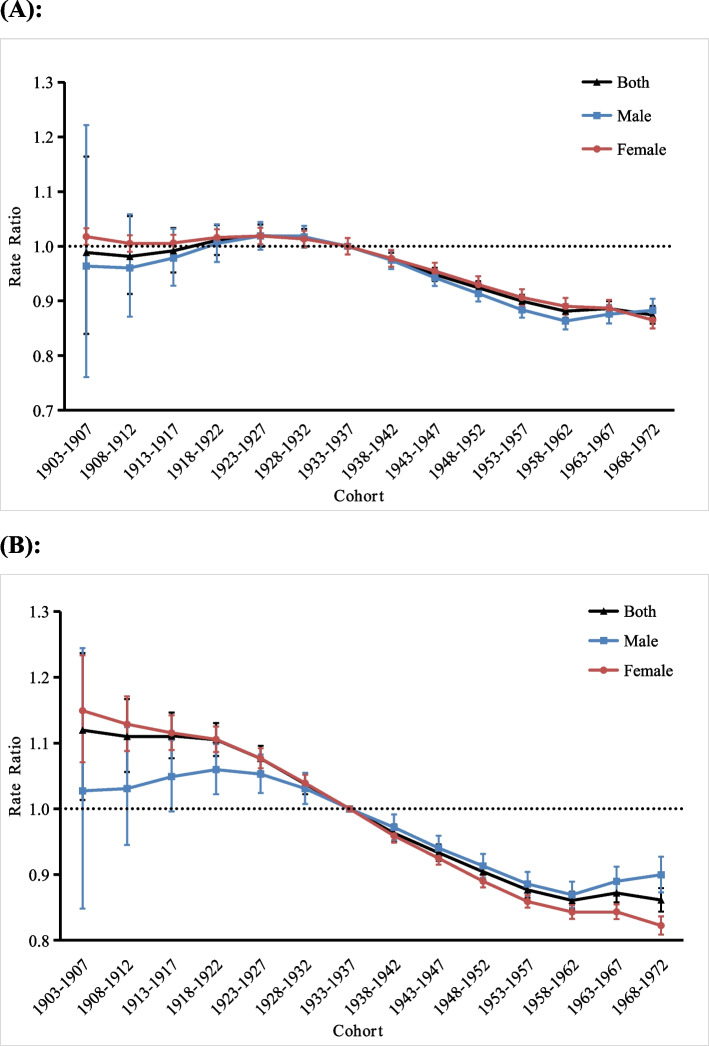
Cohort rate ratios (RR) of the incidence of and disability-adjusted life years (DALY) rate for anxiety disorders in middle-aged and older adults in China. **a** incidence, (**b**) DALY rate. The ratio of age-specific rates in each cohort relative to reference cohort (1933–1937) and the corresponding 95% confidence intervals (*note:* some of them were too narrow to be shown in the figure). The black dash lines indicate an RR = 1

Figure 5 shows the cohort RRs of the incidence and DALY rates of anxiety disorders in middle-aged and older adults in China. As the cohort effects of incidence showed (Fig. 5a), the cohort RRs in both genders, men, and women decreased from the 1903–1907 birth cohort to the 1908–1912 birth cohort and increased from the 1908–1912 birth cohort to the 1923–1927 birth cohort. The risk of incidence reached a maximum in the 1923–1927 birth cohort [both genders RR (95% CI) = 1.02 (1.00, 1.04), male RR (95% CI) = 1.02 (0.99, 1.04), and female RR (95% CI) = 1.02 (1.00, 1.04]). However, subsequently, for both genders, the cohort RRs gradually decreased from the 1923–1927 birth cohort to the 1958–1962 birth cohort, then increased in the 1963–1967 birth cohort, and finally decreased again in the 1968–1972 birth cohort. For men, the cohort RRs decreased from the 1923–1927 birth cohort to the 1958–1962 birth cohort and increased from the 1958–1962 birth cohort to the 1968–1972 birth cohort. For women, the cohort RRs showed a monotonic decreasing trend from the 1923–1927 birth cohort to the 1968–1972 birth cohort. From the 1903–1907 birth cohort to the 1968–1972 birth cohort, the risk of incidence decreased by 12.1, 8.3, and 14.7% in both genders, men, and women, respectively.

Regarding the DALY rate (Fig. 5b), the cohort RRs in both genders increased only from the 1908–1912 birth cohort to the 1913–1917 birth cohort and from the 1958–1962 birth cohort to the 1963–1967 birth cohort, with the rest showing a decreasing trend. The highest DALY risk in both genders was in the 1903–1907 birth cohort [RR (95% CI) = 1.12 (1.01, 1.24]), and the lowest was in the 1958–1962 birth cohort [RR (95% CI) = 0.86 (0.85, 0.87)]. For men, the cohort RRs showed a slow increasing trend for the birth cohorts between 1903 and 1922, followed by a decrease in the 1958–1962 birth cohort and finally an increase in the 1968–1972 birth cohort. For women, the cohort RRs only increased slightly from to the 1958–1962 birth cohort to the 1963–1967 birth cohort, with the rest showing a decreasing trend. From the 1903–1907 birth cohort to the 1968–1972 birth cohort, the risk of DALYs decreased by 23.2, 12.6, and 28.7% in both genders, men, and women, respectively.

## Discussion

The results of this study showed that the incidence of DALY rate for anxiety disorders in middle-aged and older adults in China decreased slightly from 1990 to 2019. Early recognition and treatment of anxiety disorders is a possible reason for this trend [26] (Rosen et al., 2022). China has developed rapidly over the past 30 years, and with continuous improvements in medical care and knowledge, people have been able to avoid the occurrence and development of anxiety disorders by accessing timely and effective treatment after the early appearance of symptoms. Besides, with the deepening of scientific research, new paradigms and interventions for the resilience and successful aging have been gradually explored and applied to promote the mental health and quality of life in middle-aged and older adults, which reduces the development of mental problems such as anxiety and depression [27]. Although anxiety disorders in middle-aged and older adults have decreased overall in the past 30 years, they have increased in the oldest population, which may be related to their fear of death [28] (Iverach et al., 2014). However, with increasing social pressure [29] (Levin et al., 2019) and an aging population [30] (Xiao et al., 2016), the incidence of and DALY rate for anxiety disorders in middle-aged and older adults in China showed an increasing trend over the last 5 years. This trend suggests that the burden of disease of anxiety disorders on the middle-aged and older adult population in China is not improving and should not be ignored. Therefore, more attention should be paid to the prevention and control of anxiety disorders. In addition, owing to the large population base in China, the absolute disease burden caused by anxiety disorders cannot be underestimated. Therefore, this study explored the effects on the incidence of and DALY rate for anxiety disorders from the perspective of demographic characteristics. This study also fitted the APC model in order further investigate the aetiology of anxiety disorders in middle-aged and older adults, as well as to provide scientific information for the formulation of effective prevention and treatment measures.

In this study, we found significant differences by gender in the disease burden of anxiety disorders in middle-aged and older adults in China, with women having higher ASIR and ASDR than men. This is the result of a combination of biological and psychosocial factors. Among biological factors, sex-related traits, such as genetics and sex hormones, are the main mediators of the differences between men and women in neuropsychiatric disorders. In addition, some pathophysiological pathways (e.g., the sex-microbiota-gut-brain axis) may also explain these differences [31–33] (Shobeiri et al., 2022; Farhane-Medina et al., 2022; Pavlidi et al., 2022). Regarding psychological factors, women tend to be more sensitive than men when faced with adverse events or stress, making them more psychologically prone to negative perceptions such as self-accusation and self-abasement, which increases the risk of anxiety disorders [34] (Vasiliadis et al., 2020). Regarding social factors, sex inequality remains, and women have a lower socioeconomic status than men. Women need to bear heavier stress, making them more prone to anxiety [35] (Heise et al., 2019).

In the APC model, regarding the age effects of anxiety disorders, the risk of incidence and DALY rate in Chinese men tended to increase with age in middle-age and decrease in old age, whereas in women, the incidence and DALY rate both tended to decrease with age in middle and old age. Previous studies have also shown that anxiety disorders peak in middle age and tend to decline in old age [36]. The decline in the incidence of and DALY rate for anxiety disorders in middle-aged and older adults may be due to the early onset of anxiety disorders. It has been shown that the age of onset of anxiety disorders is young, with a mean of 21.3 years [37], and that for about 99% of the population, the first onset of anxiety disorders occurs before the age of 65 years [38]. The rising risk of anxiety disorders in middle-aged men may be related to retirement. Before retirement, which generally occurs at age 50 for women and at a relatively later age of 60 years for men in China, the risks of stress and anxiety associated with approaching retirement, such as shifts in social roles, changes in life rhythms, and loss of financial resources, increase with age [39].

With the exception of a slight increase from 1990 to 1994 to 1995–1999, the incidence of and DALY rate for anxiety disorders for both genders in middle-aged and older adults in China showed a decreasing trend, which may be related to socioeconomic development and improved mental health services. Studies have shown that the risk of anxiety disorders increases at lower economic levels [40]. Over the past three decades, China has experienced rapid economic development and, as a result, the problem of anxiety disorders is likely to improve. Moreover, in response to active aging in the twenty-first century, a series of national and local policies have been developed to address geriatric mental disorders, and financial investments have been increased to promote the development of geriatric mental health services [41]. The decline in the period effects of anxiety disorders in middle-aged and older adults has been attributed to results achieved in recent years in mental health efforts. For different genders, the incidence of and DALY rate for anxiety disorders in men and women show a “scissor-shaped” trend with the period. We have not found any studies to explain this phenomenon; however, we believe that it may be related to the shift in the social status of both genders [42]. In recent years, the economic, political, and marital rights of women have increased, and the division of social roles has gradually changed to a “shared burden by both genders” in China [43]. The social stress, as well as the incidence of and DALY rate for anxiety disorders decreased in women but increased in men; therefore, there was an opposite trend in terms of the period effects between men and women.

Cohort effects showed that the incidence of and DALY rate for anxiety disorders would be lower in the later birth cohort in middle-aged and older adults in China, which may also be related to the development of mental health services. In addition, the later birth cohort tends to have a higher level of cognitive reserve, influenced by the level of education and occupational experience, which prevents cognitive decline and creates an inquisitiveness and a strong desire for knowledge about the use of technological devices and digital tools in middle-aged and older adults. It contributes to the promotion of active life in middle-aged and older adults and prevents them from being excluded from important communications and social contacts due to the lack of access to digital devices, thus reducing the sense of loneliness and anxiety in middle-aged and older adults [44]. However, the cohort RRs showed an increasing trend before 1927, which may have been associated with social unrest at that time. People in these cohorts were born during a period of revolutionary resistance and social unrest in China, which can cause stress in all aspects of life [45] and affect this population throughout their lives [46], resulting in an increased risk of anxiety. However, both the recent period and birth cohorts showed a possible unfavourable trend in period and cohort effects in the future. The decline in the recent period and cohort RRs slowed down, and even the RRs showed an increasing trend in men, suggesting that the problem of anxiety disorders in middle-aged and older adults in China cannot be ignored.

## Conclusion

Our research showed that the incidence of and DALY rate for anxiety disorders in middle-aged and older adults in China decreased slightly over the past 30 years and that both the incidence and DALY rate were higher in women than in men. Using the APC model, we found that the incidence of and DALY rate for anxiety disorders decreased in middle-aged and younger older adults but increased slightly in the oldest population, and that the incidence and DALY rate showed a decreasing trend with age. The incidence and DALY rate tended to decrease first and then increase with time in men, while they tended to increase first and then decrease in women. Cohort effects showed that the total incidence of anxiety disorders tended to increase first and then decrease, while the DALY rate tended to decrease monotonically.

In summary, the disease burden of anxiety disorders in middle-aged and older adults in China has declined over the past 30 years; however, the ASDR, ASDR, period effects, and cohort effects have shown unfavourable trends. Therefore, attention should be paid to the prevention and treatment of anxiety disorders in middle-aged and older Chinese adults. First, we should actively promote the construction of a high-level mental health service system, accelerate mental health legislation, and increase the supply of human and material mental health resources. Second, we should focus on high-risk groups and pay more attention to anxiety disorders in middle-aged and older women, while not ignoring the harm of anxiety disorders in men, especially in middle-aged males. Finally, we should insist on the principle of prevention, first, and then combine prevention and treatment. Public education regarding anxiety disorders should be promoted in addition to regular screenings in order to achieve early identification and treatment of anxiety disorders.

## Data Availability

Publicly available datasets were analysed in this study. These data are available at http://ghdx.healthdata.org/gbd-2019. After registering on the GDP official website and explaining the research intention, relevant data was obtained. We ensure that the data used in the manuscript is completely consistent with the original data in GDP. We have only conducted necessary sorting and analysis of these raw data according to the research needs.

## Abbreviations

GDB: the global disease burden
DALYs: Disability-adjusted life years
